# Improving neural machine translation for low resource languages through non-parallel corpora: a case study of Egyptian dialect to modern standard Arabic translation

**DOI:** 10.1038/s41598-023-51090-4

**Published:** 2024-01-27

**Authors:** Mohamed Atta Faheem, Khaled Tawfik Wassif, Hanaa Bayomi, Sherif Mahdy Abdou

**Affiliations:** 1 Department of Computer Science, Faculty of Computers and Artificial Intelligence, Cairo University, Cairo, Egypt; 2 Department of Information Technology, Faculty of Computers and Artificial Intelligence, Cairo University, Cairo, Egypt

**Keywords:** Mathematics and computing, Computer science, Information technology, Scientific data

## Abstract

Machine translation for low-resource languages poses significant challenges, primarily due to the limited availability of data. In recent years, unsupervised learning has emerged as a promising approach to overcome this issue by aiming to learn translations between languages without depending on parallel data. A wide range of methods have been proposed in the literature to address this complex problem. This paper presents an in-depth investigation of semi-supervised neural machine translation specifically focusing on translating Arabic dialects, particularly Egyptian, to Modern Standard Arabic. The study employs two distinct datasets: one parallel dataset containing aligned sentences in both dialects, and a monolingual dataset where the source dialect is not directly connected to the target language in the training data. Three different translation systems are explored in this study. The first is an attention-based sequence-to-sequence model that benefits from the shared vocabulary between the Egyptian dialect and Modern Arabic to learn word embeddings. The second is an unsupervised transformer model that depends solely on monolingual data, without any parallel data. The third system starts with the parallel dataset for an initial supervised learning phase and then incorporates the monolingual data during the training process.

## Introduction

A machine translation system as in^1^ uses input text in one language to translate that text into another language automatically. Researchers have played around with various content granularities, such as sentences, paragraphs, papers, and diverse material categories, such as text and audio. Only text-based sentence-level MT (Machine Translation) is of interest to us in this investigation.

This paper presents a novel investigation into the application of semi-supervised neural machine translation for low-resource languages, specifically focusing on the translation of Egyptian dialects to Modern Standard Arabic. Contrary to many existing methods, our work leverages the potentials of unsupervised learning to overcome the significant challenge of limited data availability associated with low-resource languages. We delve into the exploration of three different translation systems, each with its unique approach and benefits.

The first system employs an attention-based sequence-to-sequence model, utilizing the shared vocabulary between the Egyptian dialect and Modern Arabic to learn word embeddings. The second system takes a completely unsupervised approach, relying solely on monolingual data, without any parallel data. The third system is a fusion of the two, starting with the parallel dataset for an initial supervised learning phase, and then incorporating the monolingual data during the training process.

Our approach is specifically designed to handle the unique linguistic complexities associated with Arabic languages, as outlined in “Standard Arabic and dialectal Arabic varieties” Section. We address the issues of word concatenation, character repetition for emphasis, lexical differences between Arabic dialects and MSA (Modern Standard Arabic), and the lack of standard orthography in dialects.

There are common types of machine translation algorithms like below,

- A rule-based^2^ also known as a knowledge-based machine translation system converts input text into its equivalents by applying linguistic knowledge of the source and target languages in the form of rules.
- On the other hand, statistical machine translation^3^ creates statistical models from a set of data (usually in the form of a sentence-aligned parallel corpus). These models are later used to translate text from the source language to the target language. Machine translation is currently experiencing the deep learning wave. On language pairs like French–English and German-English, many intriguing network architectures have been presented and have performed significantly better than the ones that preceded them. The concern is that these models need a huge amount of parallel data.
- *Neural Machine Translation (NMT)* NMT^4^ is a deep learning approach to machine translation that uses neural networks to model the complex relationships between source and target languages. NMT has been shown to outperform traditional SMT methods in terms of the accuracy and fluency of the translated text.
- *Unsupervised Machine Translation (UMT)* UMT^5^ is an innovative approach to machine translation that distinguishes itself by not requiring parallel corpora for training. Instead, UMT models use techniques like cross-lingual word embedding to learn how to translate text in an unsupervised manner.
- *Semi-supervised Machine Translation* This approach leverages both labeled (parallel corpora) and unlabeled data (monolingual corpora) in the source and target languages, thereby combining aspects of both supervised (NMT) and unsupervised (UMT) methods. It is designed to be flexible and adaptable, capable of making the most out of available resources.

### Standard Arabic and dialectal Arabic varieties.

As in^5^, Today, Arabic is by far the most commonly used Afro-Asiatic language. Arabic is a Central Semitic language that dates back to the Iron Age. With up to 422 million speakers worldwide, 290 million of whom are native Arabic speakers, modern Arabic is a mix of dialects. Arabic is the fifth most spoken language overall, both in terms of native speakers and total speakers.

Classical Arabic and Modern Standard Arabic are the two recognized standard dialects of the Arabic language. The Quran is written in classical Arabic. The early Islamic era saw significant spelling alterations, including adding dots to separate letters and diacritics to denote short vowels. Modern Standard Arabic (MSA), one of the six official languages of the United Nations, was created from Classical Arabic in the early nineteenth century to become the standardized and academic variant of Arabic.

Colloquial Arabic, another name for dialectal Arabic, describes a variety of regional dialects that developed from Classical Arabic, sometimes separately from one another. They have been greatly impacted by the native tongues that predated Arab conquest and coexisted with Arabic afterward. For instance, Levantine, Egyptian, and Moroccan cultures were affected by Aramaic and Syrian, Coptic, and Berber, respectively. Additionally, because most of these territories were occupied by foreign nations, the dialects of Turkish, French, English, Italian, and Spanish were all impacted to differing degrees. These influences caused significant differences across Arabic dialects, to the point that some varieties—like the Maghrebi dialects, for instance, are unrecognizable to a speaker of an Egyptian dialect*.*

### Challenges in Arabic dialects

Current Arabic is a collection of varieties, which include Modern Standard Arabic (MSA), which has a standard orthography and is used in formal contexts, and Dialectal Arabic (DA), which also are commonly used unofficially and have a growing presence on the internet.

#### Overview of Arabic Varieties

Arab dialects differ from one Arab country to another, and even within the same country, significant differences exist^6^; There are huge differences between Arabic dialects, to the point where some varieties, such as Maghrebi dialects, are unintelligible to Egyptian dialect speakers.

Many difficulties are shared between Arabic dialects and Modern Standard Arabic. complex words like The repeating linguistic practicing of concatenating and dropping letters to combine multiple words, as in the case of the word ‘مبيجلهاش’^7^ and the use of emoticons and character repetition for emphasis ‘ادعووولي’^7^. Also, several words found in Arabic dialects do not have the same meaning as those found in MSA. Like ‘مش’, ‘بلاش’, ‘تاتو’.

#### Linguistic challenges

Due to the morphologically rich language of Arabic, natural language processing of Arabic faces many challenges^8^, There are also differences between dialects and Arabic because there is no written set of grammar rules. As an example, there is no standard orthography in dialects, so every dialect spells the same word differently like (ماء, مويه, ميه) for water.

The substitution of specific letters. For example, the interdental sound of the letter ث is frequently replaced by either ت or س, as in كثير “much,” and the glottal stop is reduced to a glide, as in جائز “possible.” turned to جايز ^7^

Also, the ambiguity caused by the use of diacritical marks, known as Tashkil in Arabic, changes the meaning of the same word. Another feature is a misspelling in dialect as they spell differently in MSA; for example, the word gold can be written as (ذهب) in MSA and as (دهب) in EGY^9^.

This paper is organized as follows: “Related work” Section describes, in brief, the related work of translation of Egyptian Arabic to modern Arabic, it discusses different translation algorithms. “Description of our Egyptian dialect—standard Arabic datasets collection method” Section describes the data used in our translation models and how we collected it. “Models Architectures” Section describes the different deep learning architectures we have used in our experiments. “Defining measurement tool—Bleu Score” Section describes the metric used to measure our model’s accuracy (Bleu). “Results” Section describes the results of the experiments and how our machine translation is good in our problem and the tools we used in the experiments. “Conclusion” Section describes the conclusion of our work and our expectation for the future.

## Related work

Machine translation is a challenging task especially unsupervised learning where the goal is to learn to translate between languages without any parallel data. While the field is relatively new, there have been several works exploring different approaches to address this problem. Here are some of the related works in Arabic Dialectal machine translation and unsupervised machine translation related work:

### Arabic machine translation related work

In this paper^9^, They are proposing algorithms that try to overcome the limitations of low-resource languages and apply them to translate Egyptian dialects (EGY) to Modern Standard Arabic (MSA). For both MSA and EGY, monolingual corpora were collected, and a relatively small parallel language pair set was created to train the models. Because it requires monolingual data rather than a parallel corpus, the proposed method employs Word embedding. To create word vectors, both Continuous Bag of Words and Skip-gram were used, Word2vec by^10^. Using a four-fold cross-validation approach, the proposed method was validated on four different datasets with the highest score of Egyptian Arabic to standard Arabic 25.35 bleu score.

This paper^11^ recommended a generic method for converting an Egyptian colloquial (Egyptian Dialect) Arabic sentence to a vocalized MSA sentence. They were using a statistical approach to tokenize and tag Arabic sentences automatically. As well as a rule-based approach for producing the target diacriticized MSA sentence.

The work was assessed using a dataset of 1 K Egyptian dialect sentences (800 sentences for training and 200 sentences for testing). Converting Egyptian Colloquial Arabic words into their corresponding MSA words yielded an accuracy of 88 percent.

In this paper^12^ They presented a rule-based strategy for generating Colloquial Egyptian Arabic from modern standard Arabic, and they provide an application case to the Part-Of-Speech (POS) tagging task, for which the accuracy was improved from 73.24 percent to 86.84 percent on unobserved CEA text, and the percentage of Out-Of-Vocabulary (OOV) words was decreased from 28.98 to 16.66%.

This paper^13^ presents ELISSA, ELISSA is a DA-to-MSA machine translation (MT) system. To generate MSA paraphrases of DA sentences, ELISSA employs a rule-based approach that relies on morphological analysis, transfer rules, and dictionaries, in addition to language models. When using MSA NLP tools, ELISSA can be used as a general preprocessor for DA. It was shown that 93% of MSA sentences.

Produced by Elissa were correct. Elissa was used for pivoting through MSA in a dialect-English MT system, and the BLEU score improved by 0.6 to 1.4%.

In this paper^14^ A rule-based method based on a linguistic model was employed to translate the Moroccan dialect into MSA. The system is based on morphological analysis using the Alkhalil morphological analyzer^15^, which has been modified and extended to include Moroccan dialect affixes, as well as a bilingual dictionary (created using situations from television shows and data gathered from the internet). The text is examined and divided into annotated dialect units following an identification process that separates dialectal data from MSA. These outputs are connected into one or more MSA corresponding units by utilizing the bilingual dictionary. The most fluid MSA sentences are created by passing the generated MSA phrases through a language model.

#### Supeervised and unsupervised MT related work

In this paper^16^ the authors outline a research project that aims to tackle the challenge of machine translation for Arabic dialects. The authors draw a distinction between rule-based and statistical machine translation, and highlight the issue of dialects being less effectively translated compared to standard or modern Arabic. To address this problem, they introduce the Idea of “automatic standardization,” which uses machine translation methods to generate standard Arabic text from a dialect input. The authors opt to use statistical models for this approach, as developing linguistic rules for each dialect is challenging. The ultimate goal of the study is to combine automatic standardization software with automatic translation software to produce high-quality translations of Arabic dialects. Additionally, the authors suggest that this could have educational implications, such as facilitating comprehension of various Arabic dialects by transforming dialectal text into standard Arabic.

In the realm of unsupervised learning for machine translation, significant strides have been made. For instance,

This paper^17^ further advanced the field of unsupervised machine translation by proposing a method that exclusively uses monolingual corpora to learn translations between languages. Rather than relying on parallel corpora, their approach leverages monolingual data, which is often more abundant, particularly for low-resource languages. This method underscores the potential of monolingual corpora in enhancing machine translation for languages with limited parallel data.

In the domain of unsupervised learning for machine translation, novel methodologies have been proposed to address the challenges associated with low-resource languages. A significant contribution in this field is the work in this paper^18^, who put forward an unsupervised neural machine translation approach that capitalizes on weight sharing. This innovative technique does not depend on parallel data, which is often scarce for low-resource languages, but instead learns translations between languages by sharing weights across different layers of the neural network. This approach demonstrates the potential of unsupervised learning techniques in reducing the data requirements and enhancing the scalability of machine translation systems.

### Human participation

Human participants were involved in the data collection process for our study. Using a Google Form, they provided a few translations examples from Egyptian Arabic to Modern Standard Arabic. Following this, another individuals reviewed these translations for accuracy, making necessary corrections. This human involvement ensured the high quality and reliability of our collected data, these collected data is around 5% of human participants the rest is by me.

## Description of our Egyptian dialect—standard Arabic datasets collection method

We have collected two datasets the first one is the two monolingual Egyptian Arabic and standard Arabic datasets,

The second one is the parallel corpus of Egyptian Arabic to standard Arabic, in this section we will explain how we collected the datasets.

### Egyptian dialect and standard Arabic monolingual corpora

We started collecting Egyptian Arabic dialect data from several different sources on the Internet, such as the social networking site Fatakat, Facebook, and Twitter, and we were able to collect more than 15 million sentences and perhaps more from social networking sites, each sentence ranging from five words to 50 words.

**Figure 1:**
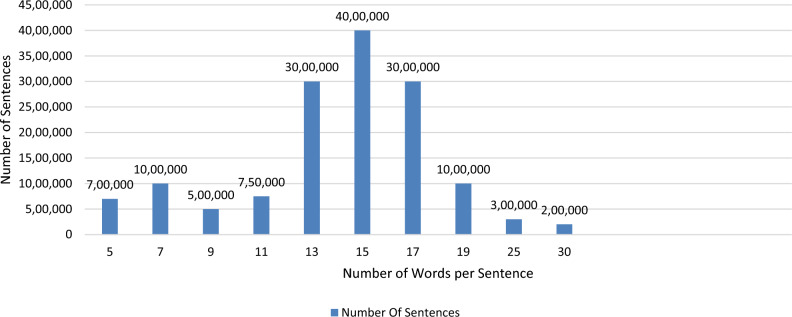
Number of sentences of combined Egyptian and Standard Arabic.

Also, we have begun to collect data from the modern Arabic language from official pages such as national newspapers, for example, the Al-Youm Al-Sabaa website, and from Wikipedia documents in Standard Arabic. We have been able to collect up to 20 million sentences, and each sentence ranges from ten to 50 words More statistics are shown in Fig. 1. That introduce information about the dataset used in our experiments.

### Egyptian dialect and standard Arabic parallel corpus

We have collected our parallel data set of Egyptian dialect and modern Arabic, and we have translated more than 40,000 Egyptian colloquial sentences into modern Arabic using social communication methods and our friends, and Arabic language teachers to help us translate these sentences. To speed up the translation process from Egyptian dialect to Standard Arabic in the unsupervised setting. A few examples are shown in Table 1 Above.

**Table 1:** Egyptian to standard Arabic sample pairs.

Egyptian sentence	Standard Arabic version
احنا بقلنا يومين ع الموضوع ده وبنقنعهم ومافيش فايده والله زهقت منهم	نحن منذ يومين في نفس هذا الموضوع ونقنعهم ولا فائدة والله مللت منهم
سعات بحس اني عاوزه امشي اطبطب على الناس و احضنهم حقيقي مفيش انسان يستاهل انه يحس انه وحيد او ان خلاص كل حاجه انتهت بالنسباله	أحيانا أشعر أنني أريد أن أسير أطبطب على الناس وأحضنهم حقيقة لا يوجد إنسان يستحق أن يشعر بأنه وحيد أو أن كل شيء انتهى بالنسبة له
حب نفسك جدا, واعرف انك ماتتعوضش, واعرف ان شكلك مميز وصوتك وطريقة كلامك وكمان طريقة نقاشك, والعقل والجنان اللي فيك ولما بتقسى ولما بتكون حنين على اللي قدامك, وخلي ده ميبقاش موضع غرور لا ده يبقى عن اقتناع, انت لا قليل ولا رخيص في حياة حد, انت غالي جدا وعزيز جدا واحلى مما تتخي	حب نفسك جدا, واعرف أنك لا تعوض, واعرف أن شكلك مميز وصوتك وطريقة كلامك وأيضًا طريقة نقاشك, والعقل والجنون الذي فيك ولما بتقسو ولما تكون حنين على الذي أمامك, ولاتجعل هذا يكون موضع غرور لا هذا يكون عن اقتناع, أنت لست قليل ولا رخيص في حياة أحد, أنت غالي جدا وعزيز جدا وأحلى مما تتخيل
واحد امريكي وواحد عربي الامريكي قال للعربي. احنا صنعنا صواريخ ووصلنا القمر العربي بصلو. وقالو. القمر فيه ستات وخمره الامريكي قالو. لا العربي قالو. لو كان فيه ستات وخمره. كنا طلعنالو قبلكم. واحد فوق واحد	شخص أمريكي وآخر عربي قال الأمريكي للعربي نحن صنعنا صواريخ ووصلنا إلى القمر نظر إليه العربي وقال له هل يوجد بالقمر نساء وخمر أجابه الأمريكي لا فقال له العربي لو وجد هناك نساء وخمر لسبقناكم إليه . ربحت الجولة
ايه انواع اللعب المناسبة لكل مرحله عمرية? في اهالي كتير بيغلطوا في انهم يشتروا كميات لعب كثيرة جدا للطفل من غير ما يركزوا في المرحلة العمرية او اهتمامات الطفل او المهارات اللي محتاجة تتنمى عنده. اللعب الحسي مهم انك تبدايه لابنك او بنتك من اول سن 9 شهور	ما أنواع الألعاب المناسبة لكل مرحلة عمرية ؟ هناك الكثير من الأهالي تخطئ عندما يشترون لأطفال كميات هائلة من الألعاب بدون إبداء اهتمام لاهتمامات الطفل أو المرحلة العمرية أو المهارات التي يحتاج إلى تنميتها . ومن المهم أن تبدئين بممارسة الألعاب الحسية مع ابنك أو ابنتك من أول سن التسع شهور

## Models architectures

In this section, we will present our Egyptian—Standard Arabic machine translation methods, the first system is the Supervised Sequence-to-Sequence RNN with node type LSTM^19^) Encoder–Decoder with Attention Mechanism^20^. (RNN), a class of artificial neural networks where connections between nodes form a directed graph along a temporal sequence. The second mechanism is an Unsupervised Encoder Decoder^21^. The last one is the combination of Supervised and Unsupervised mechanisms, a parallel corpus of Egyptian Arabic to standard Arabic is used to boost the quality of the model then continue with the unsupervised settings.

In our research, we employ a Transformer-based Neural Machine Translation (NMT) model, renowned for its proficiency in handling machine translation tasks, notably for low-resource languages. The architecture includes a three-layer encoder and decoder, capitalizing on an attention mechanism to concentrate on distinct segments of the input sentence while formulating the output sentence.

The chosen hyperparameters include an embedding dimension of 512, a standard size for Transformer models. To enhance the model's generalization ability and reduce its complexity, parameters between the encoder and decoder's initial three layers are shared. This sharing extends to language embeddings, output embeddings, encoder–decoder embeddings, and decoder pre-output embeddings.

For training, we utilize all available sentences in both monolingual and parallel datasets. We apply regularization via word shuffling, dropping, and blanking during training. Optimization is achieved using Adam with a learning rate of 0.0001, and cross-entropy loss weights are adjusted throughout training. We conduct our training with a batch size of 16 and limit each epoch to 500,000 iterations. Additionally, we maintain a maximum sentence length restriction of 100 tokens during training. We trust that these methodological choices and hyperparameters provide a comprehensive understanding of our approach and serve as a valuable reference for future work in this area.

A detailed description of every mechanism in the rest of the paper.

### Supervised sequence-to-sequence LSTM encoder–decoder with attention for Egyptian-standard Arabic translation

The central concept of LSTM^19^ is the use of a special memory to control how much information is passed or blocked from the recurrent neural unit. The memory cell is made up of three gates (input gate, memory gate, and output gate)^22^.

**Figure 2:**
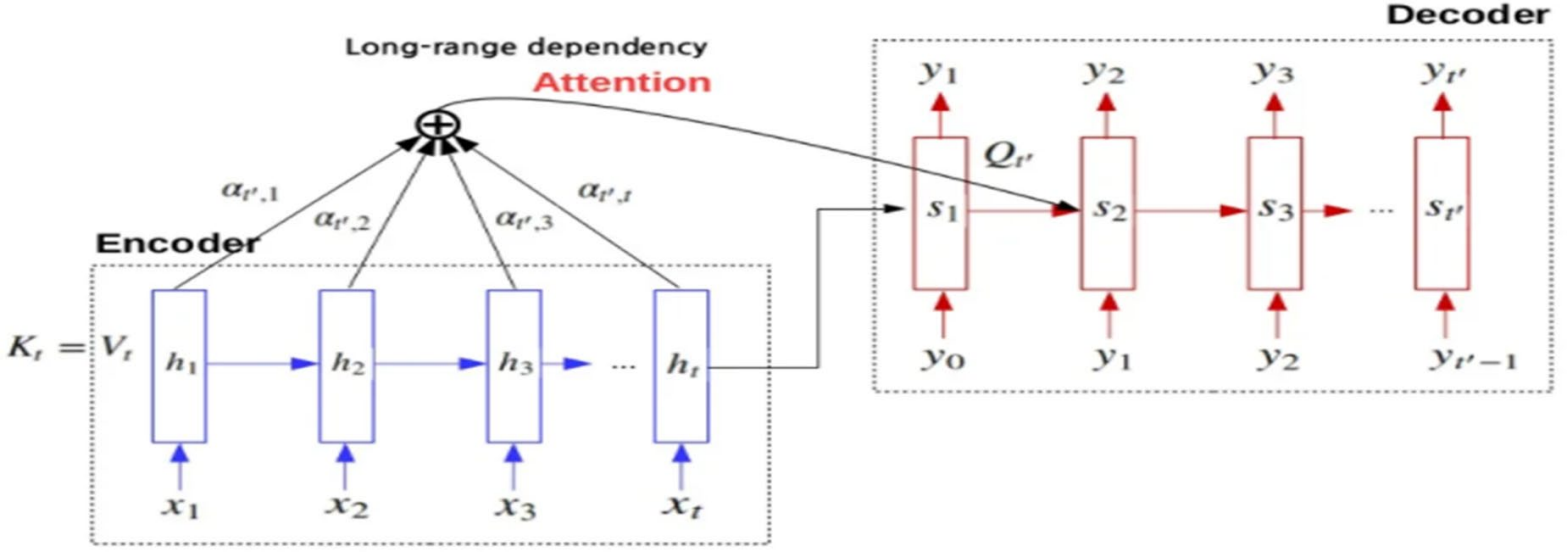
RNN encoder–decoder with an Attention mechanism.

With the problem of bias of context vector in RNN to the last words in the sentences. And the vanishing problem of the long words, the Attention mechanism^4^ appears As depicted in Fig. 2 to solve this problem and help the decoder part to predict the next word in long sequences as the decoder utilizes the context vector of the encoder (the encoded vector) and the weighted sum of the hidden states of the words in the source language.

We trained the LSTM^19^ model (normal RNN) with 4 layers in the encoder and 4 layers in the decoder with a learning rate of 0.001, batch size of 16, and the dimensionality of embedding is 512 with the same configuration in several hidden nodes in the hidden layers.

The same setting was applied with 300-word embedding and 300 hidden neurons in every hidden layer.

### Unsupervised encoder–decoder approach for Egyptian-standard Arabic translation

According to^21^ They proposed a translation model comprised of an encoder and a decoder, with the encoder responsible for encoding source and target sentences to a latent space and the decoder responsible for decoding from the latent space to the source or target domain. In addition, they use a single encoder and decoder for both domains^20^. The only difference when applying these modules to different languages is the choice of lookup tables as we depend on BPE byte pair encoding according to^23^ which has two major benefits: they reduce the size of the vocabulary and eliminate the presence of unknown words in the output translation. Second, rather than learning an explicit mapping between BPEs in the source and target languages, we define BPE tokens by.

Processing both monolingual corpora concurrently. If two languages are related, they will naturally share a large number of BPE tokens.

This section details an unsupervised machine translation algorithm designed to facilitate translation between Egyptian and Standard Arabic, particularly useful where parallel corpora are limited. The algorithm utilizes an Encoder–Decoder model combined with Byte Pair Encoding (BPE) to manage the vocabulary size and eliminate unknown words, while processing both monolingual corpora concurrently to leverage shared linguistic features.

**Figure 3:**
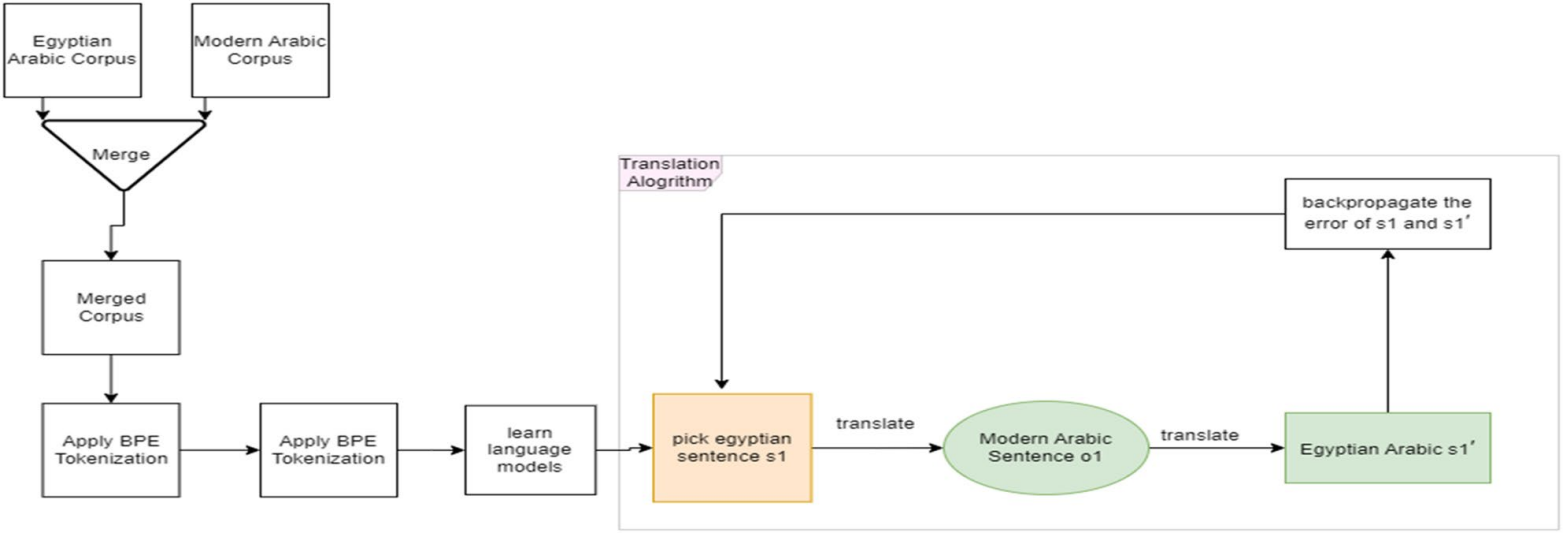
The unsupervised learning part begin by joining the corpus Then preprocessing it after that BPE Tokenization applied At the end the process of unsupervised learning begins.

The algorithm of training unsupervised machine translation as in Fig. 3 below,

1. Append the monolingual corpora to one corpus.
2. Apply BPE tokenization on the resulting corpus.
3. On the same corpus, learn token embedding as in^10^.
4. Learn two language models the first one to translate.

Egyptian Arabic sentence from a noisy Egyptian sentence, the second to translate a modern Arabic sentence from the noisy version of it using auto-encoder architecture as described in the below paragraph, the encoder encodes a noisy version of a sentence and the decoder decodes the output of the encoder to the original sentence.

1. Using the learned language models, learn two initial.

Poor translation models one From Egyptian to Arabic.

And the other from Arabic to Egyptian.

(2) Iterate over the monolingual sentences of the two corpora

- Pick a random Egyptian sentence from the monolingual Egyptian corpus.
- Generate Arabic sentences from Egyptian sentences using the initial Egyptian- > Arabic translation model.
- Pick a random Arabic sentence from the monolingual Arabic corpus.
- Generate an Egyptian sentence from the picked Arabic sentence using the initial Arabic- > Egyptian Translation model.
- Train the translation models (Egyptian- > Arabic and Arabic- > Egyptian) using the generated sentences from the previous translation step.

Autoencoders are a type of artificial neural network used for learning efficient codings of input data as in^24^, typically for the purpose of dimensionality reduction or denoising. They work by encoding input data into a compressed representation, and then decoding this representation back into the original format. The aim is to minimize the difference between the original input and the reconstruction, often using a loss function that measures this difference.

In the context of your unsupervised machine translation model, the autoencoder is used to learn language models for Egyptian Arabic and Standard Arabic. The encoder part of the autoencoder takes a noisy sentence as input and encodes it into a latent space. The decoder part then attempts to reconstruct the original sentence from this latent representation. Through this process, the autoencoder learns a mapping from sentences to a compressed representation and back that captures the underlying structure of the language. The learned representations can then be used as a basis for translation between the two languages.

### Hybrid approach: Combining supervised and unsupervised mechanisms for Egyptian-standard Arabic translations

The main contribution of this work is the use of the unsupervised technique with the supervised one.

A subset of machine learning is semi-supervised learning, It refers to a learning problem (and algorithms created for the topic) where a model must learn from a limited number of labeled instances and a large number of unlabeled examples to generate predictions about new examples.

It is directly applicable to a variety of real-world problems^25^ when labeled data production is very expensive and only a small number of labeled training points are accessible, but a big number of unlabeled points are provided.

We first speed the learning of the model with labeled data (40 thousand sentence pair) after that the model start unsupervised learning as in Fig. 3.

## Defining measurement tool—Bleu score

Bilingual evaluation Understudy is what BLEU^26^ stands for. It is a metric used to assess the text quality produced by a machine by contrasting it with a reference text that was intended to be produced. Typically, a manual evaluator or a translator creates the reference text^26^.

Machine translation reviews by humans are accurate but expensive. Human evaluations might take months to complete and require disposable human labor.

We decided to use BLEU Score for evaluating automatic machine translation that is quick, affordable, and language-independent, has a strong correlation with human review, and has a low marginal cost per run.

As Machine Translation is one of the use cases of the BLEU Score as it determines how closely the model-generated translation adheres to the source language.

Bleu Formula as in^26^:

In the Bleu score, we calculate the number of correct predicted n-grams/Number of total predicted n-grams from 1-g through n-gram.

And give a penalty to the short predicted sentences as below in Eqs. (1) and (2).

1 $$ BLEU = BP * exp\left( {1/n * \left( {log\left( {p\_1} \right) + log\left( {p\_2} \right) + \cdots + log\left( {p\_n} \right)} \right)} \right) $$

2 $$ BP = min\left( {1, exp\left( {1 - reference\_length / output\_length} \right)} \right) $$

where BP is the brevity penalty, which is a correction factor that reduces the score for translations that are shorter than the reference translations.

N is the maximum n-gram size considered in the evaluation.

p_1, p_2, …, p_n are the n-gram precisions, which are calculated as the ratio of the number of matching n-grams in the machine-translated output to the total number of n-grams in the output.

To quantitatively evaluate our models, we used BLEU score^26^, which is a commonly used metric for machine translation. BLEU score compares the output of a machine translation system against human (reference) translations, with a higher score indicating greater correspondence. It calculates n-gram precision between the candidate and reference translations, along with a brevity penalty for shorter output. BLEU score provides an automated way to evaluate translation quality and has been shown to correlate well with human judgements. Consequently, we report BLEU scores to compare the performance of our different models.

## Results

Throughout this study, an extensive range of experiments were conducted to investigate the optimal neural machine translation (NMT) system for the Egyptian dialect of the modern standard Arabic language. Various network architectures, learning rates, and encoder–decoder configurations were explored and compared, as detailed in section IV.A, to identify the most promising parameters. Three distinct models, namely the supervised setting, unsupervised setting, and semi-supervised setting, were thoroughly examined to determine their effectiveness in handling the translation task.

The supervised setting involved training the network on a dataset consisting of 40,000 manually prepared parallel sentence pairs, covering both the Egyptian Arabic and modern standard Arabic languages. The unsupervised setting, on the other hand, relied on training the network using approximately 20 million monolingual sentences in both languages, which were sourced from websites such as Wikipedia and other online resources. The semi-supervised setting sought to combine the advantages of both supervised and unsupervised learning by balancing the need for parallel data and the demand for larger monolingual datasets.

A meticulous evaluation of the models using BLEU scores, as presented in Tables 2 and 3, revealed that the semi-supervised setting outperformed the other approaches. Furthermore, Table 4 showcases examples of the output generated by the system, comparing the translations with reference sentences to provide a better understanding of the system's performance.

**Table 2:** MT results on Egyptian—standard Arabic—300 word embedding.

Training data	RNN (LSTM) with attention	Transformers settings
Supervised Settings, 40,000 parallel sentences paired in Egy and Modern Arabic	19 Point	19.07 Point
Unsupervised Settings, 20 million sentences of monolingual corpora in both Egy and Modern Arabic	12 Point	14 Point
Supervised + Unsupervised Settings, In both datasets, the first is 40,000 parallel sentences paired in Egy and Modern Arabic, and the second one is 20 million sentences	22 Point	24 Point

**Table 3:** MT results on Egyptian—standard Arabic—512 word embedding.

Training data	RNN (LSTM) with ATTENTION	Transformers settings
Supervised Settings, 40,000 parallel sentences pair in Egy and Modern Arabic	22 Point	24 Point
Unsupervised Settings, 20 million sentences of monolingual corpora in both Egy and Modern Arabic	18.7 Point	18 Point
Supervised + Unsupervised Settings, In both datasets, the first is 40,000 parallel sentences pair in Egy and Modern Arabic, and the second one is 20 million sentences	25 Point	29.5 Point

**Table 4:** Sample of system output with a comparison of reference sentences.

Index	Egyptian	English	Reference	Generated
0	هل انت فعلا تقدر تعملها	Can you really do it?	هل تستطيع فعلها حقا	هل يمكنك حقا القيام بذلك
1	ممكن تساعدني الاقي شنطي	Can you help me find my bag?	هلا ساعدتني في ايجاد امتعتي	هل يمكنك مساعدتي في العثور علي امتعتي
2	دي احلي حاجه	This is the best thing	هذا هو اجمل شيء	هذا هو اجمل شيء
3	انت ذكي جدا يا جمال انت عارف كل حاجه	You are very smart, Gamal, you know everything	انت ذكي جدا يا جمال . كل ما تعرفه	انت ذكي جدا يا جمال اعلم انك تعرف كل شيء
4	بتعرف تسوق	Do you know how to drive?	هل يمكنك القياده	هل يمكنك القياده
5	محتاج اي مساعده	Do you need any help?	هل تريد اي مساعده	هل تحتاج اي مساعده يا صديقي
6	الصين بتسعي بكل جهدها عشان تعكس شكل النمو التقليدي اللي بيعتمد علي الصادرات ومشاريع راس المال الكبيره	China is striving hard to reverse the traditional growth model that relies on exports and large capital projects	تعمل الصين جاهده لعكس نموذج النمو التقليدي الذي يعتمد علي الصادرات والمشاريع الراسماليه الكبيره	تعمل الصين جاهده لعكس نموذج النمو التقليدي الذي يعتمد علي الصادرات والمشاريع الراسماليه الكبيره
7	لما الشخص يكون عارف انه اتخدع لازم في نفس الوقت يتاكد من تجربه الحياه الحقيقيه من جديد	When a person realizes they've been deceived, they must at the same time assure themselves of experiencing real life anew	عندما يعلم الشخص انه مخدوع يجب عليه اعاده تاكيد تجربته الحقيقيه في الحياه	عندما يتعلم الشخص انه مخدوع يجب عليه اعاده تاكيد تجربته الحقيقيه في الحياه

In light of the comprehensive analysis conducted in this research, it can be concluded that the semi-supervised approach is the most effective strategy for the development of an NMT. system specifically designed for the Egyptian dialect of the modern standard Arabic language. This finding not only contributes valuable knowledge to the field of NMT but also has the potential to significantly enhance the translation quality for this particular language pair.

## Conclusion

In this study, we have addressed the unique challenge presented by Arabic dialects, such as the Egyptian dialect, which do not possess the systematic rules found in modern standard Arabic. To overcome this issue, we explored the application of advanced deep learning techniques, aiming to investigate potential mathematical solutions through the use of deep learning methodologies. Our research involved experimenting with three distinct deep learning approaches, including supervised, unsupervised, and semi-supervised techniques. The semi-supervised method, in particular, focused on training the model with parallel corpora initially, followed by further learning using monolingual corpora.

The supervised approach involved training a model with a dataset consisting of parallel sentence pairs in both languages. The unsupervised approach involved training a model using monolingual sentences in both languages. The semi-supervised approach combined the strengths of both supervised and unsupervised learning, starting with training on parallel corpora and then further learning with monolingual corpora.

From our experiments, we found that the semi-supervised learning approach outperformed both the supervised and unsupervised approaches as per the results above Table 3. This demonstrates the effectiveness of combining both labeled (parallel corpora) and unlabeled data (monolingual corpora) in the training process. It also shows that our work successfully managed to leverage the potentials of both supervised and unsupervised learning for the task of machine translation in this context.

To mitigate the problem of out-of-vocabulary words, we incorporated byte pair encoding during the word embedding phase, ensuring a more comprehensive representation of the language. We conducted a series of experiments with various models, ultimately discovering that the semi-supervised technique consistently achieved the highest BLEU scores compared to both the supervised and unsupervised methods. This improved performance was attained by training the system on parallel corpora consisting of the Egyptian dialect and modern standard Arabic.

In terms of future work, we are determined to enhance the system's performance through two primary strategies. Firstly, we will delve into the potential of the GPT architecture, as referenced in^27^, to further improve translation quality. Secondly, we aim to expand the Egyptian dialect—modern standard Arabic dataset by incorporating a wider range of complex sentences, thereby enriching the training data available for the model. In addition to these strategies, we are interested in extending our research to include experiments on other Arabic dialects, such as Moroccan Arabic and Algerian Arabic, with the goal of broadening the scope and applicability of our findings to a more diverse set of language pairs. By pursuing these avenues, we hope to make a significant contribution to the field of neural machine translation for Arabic dialects and beyond.

## Data Availability

The datasets generated during the current study are available to download though the first author email. The source code is available from the corresponding author on reasonable request at the following link https://github.com/mohamedatta93/EGY_MSA_Translation.git. We confirm that all experimental protocols were approved by the Faculty of Computers and Artificial Intelligence—Cairo University. We confirm that all methods were carried out in accordance with relevant guidelines and regulations. We confirm that we obtained informed consent from all subjects involved in the study.
